# Book Reviews

**Published:** 1804-08-01

**Authors:** 


### CRITICAL ANALYSIS

OF THE

### RECENT PUBLICATIONS

ON THE

### DIFFERENT BRANCHES OF PHYSIC, SURGERY, AND MEDICAL PHILOSOPHY.

---

*A Treatise on Gun-Shot Wounds, which obtained the Premium given by the Royal College of Surgeons in London, for the Year* 1803, *by* T. Chevalier, 8vo. pp. 146, London, 1804.

The Author of this valuable Treatise had for some time meditated the composition of such a work, when the Royal College of Surgeons, in the year 1801, announced Gun-Shot Wounds as the subject of a Prize Dissertation.

" This induced me (says Mr. C.) to lay aside my intention, the fulfilment of which, I thought would probably be rendered unnecessary. But finding they were again proposed as a Thesis for last year, and thinking the circumstances of the times made it desirable that something should appear on the subject, I resolved to commit my ideas to writing, and to submit them, unknown, to the judgmentof the College.

" By this method I was sure of obtaining the unbiassed opinion of competent judges in the first instance; and their decision having been in my favour, I am more satisfied, than if I had merely asked the opinion of any friend, as a friend, or trusted entirely to my own.

" It may however be proper to observe, that the object I have had in view in writing this treatise, has simply been, to make a faithful and correct investigation of the characteristic phænomena of Gun-shot Wounds; to explain their effects upon indisputable principles in physiology and pathology, to point out those processes by which only nature can repair all that is reparable in such a complication of violence; and to deduce from thence that treatment by which she may be most effectually assisted in her work, and the obstacles to her performance of it in the best and safest manner, may be either prevented or removed.

" Such then having been my object, while I hope my labour will not be in vain, I am desirous that the reader should avail himself of every other assistance by which he can perfect his knowledge of the subject, and illustrate the doctrines, or supply the deficiencies he may observe in this treatise. I would particularly recommend his perusal of the small, but masterly work of Le Dran on Gun-shot Wounds, which I once had a thought of translating, and publishing, with such alterations as would comport port with our later improvements. But I soon found that these must be so numerous as to make it not worth the trouble. I cannot however withhold my praise from a treatise, which I never take up without pleasure. The work of Ravaton is excellent in its kind. Those of the late Mr. Hunter, and of Mr. John Bell on the same subject, notwithstanding their difference in some particulars, are also well worth an attentive perusal. And perhaps in these four works will be found the substance of most, if not of all, that can be collected from former writers; freed from a number of errors, and enriched with many solid and judicious remarks. Many cases however, recorded in the Memoirs of the Royal Academy of Surgery at Paris, and in other works both in French and English (not forgetting the writings of old La Motte, and Mr. Serjeant Wiseman) will be of use to the student, if the circumstances in them which are of real importance be properly discriminated from the rest, and from the obsolete theories with which they are intermixed."

Mr. C. divides his Treatise into two parts; the first is devoted to the consideration of the true nature and character of Gun-shot Wounds, in which the Author treats of the "nature of wounds in general; the nature of contusion, laceration, hæmorrhage, and fracture; the operation of extraneous substances on the living solid; and the laws by which the course and effect of bodies in motion are necessarily determined."

Mr. C. concludes his first part with the following observations.

"From what has been said, may be seen the reason of that concussion or shock, *(ebranlement)* which is given, in many instances, to the whole system, by the infliction of a gun-shot wound, and which has been remarked by the best writers on this subject, to be often attended with grave, and even alarming effects; extending not only over the injured part, but affecting the system at large. For as the resistance to the shot is afforded, not only by the texture of the injured part, but also is in part made up by the connection this has with other parts, and with the whole body, these also will therefore participate in the violence; and they will do it so much the more, in proportion as the part immediately wounded, has from its attachments, its texture, elasticity, or importance to life, a greater connection with the stability, or with the functions of the rest. Hence a shot striking against a tendon or a bone in one of the extremities, will produce a greater concussion than if it struck only against softer parts; a shot striking against a muscle in action, will produce more concussion than if it struck against the same part of the same muscle at rest; and a shot striking the head, or wounding the liver, lungs, or intestinal canal, will generally bring on an instantaneous derangement of the whole system, with which the functions of these parts are so closely connected.

"To all this must be added, an alarm and apprehension which immediately come upon the mind, which is often increased by the uncertainty uncertainty of the patient about his real state; but which, in wounds of some parts, the most determined courage is not always sufficient to withstand.

"Having thus endeavoured to analyse the phænomena of a gun-shot wound, considering it first as a complicated species of violence committed on matter variously organized, and also to explain its effects as violence committed on *living* matter; and having pointed out the processes which naturally ensue from each of these circumstances respectively, in order to shew the indications of cure which may be deduced from them, I shall now proceed to point out by what mode of treatment those indications may be most rationally and successfully pursued."

In the second part, Mr. C. delivers the treatment of gun-shot wounds, which he does, as he has before done the description of them, with great clearness and precision. But our commendation is not necessary to secure this excellent Tract an attentive perusal; the character of the Author, and the College of Surgeons have done it already.

---

*Surgical Observations, containing a Classification of Tumours, with Cases to illustrate the History of each Species; an Account of Diseases which strikingly resemble the Venereal Disease, and various Cases illustrative of different Surgical Subjects. By* John Abernethy, F. R. S. &c. &c. 8vo. pp. 263. 1804.

The importance of an arrangement of tumours leading to an accurate description of their appearance, history, and treatment, must be admitted by every one. Impressed with this idea, Mr. Abernethy has for some years past made use of the extensive opportunities the practice of St. Bartholomew's allows him to select his different specimens, to exhibit them at his lectures with an arrangement he now offers to the public at large. To this last he was prompted by a consideration of the importance of the subject, and that it could only be undertaken by men of such large opportunities, though others might comprehend it when demonstrated. A still further inducement was, that the minds of men have lately been laudably incited to the investigation of cancer, in hopes of discovering a cure, that the society instituted for that purpose have proposed certain questions which the author has attempted to answer, and lastly, as much *collateral* knowledge is required to investigate any subject with accuracy, that probably this paper may tend to point out the required distinctions, and furnish such collateral knowledge. After these preliminary observations, the author introduces his definition by a few remarks on his predecessors.

"The subject of tumours occupies a considerable space in the works of the antient writers on medicine. They seem, however, to have considered the subject rather with regard to its name than its nature; for we find a great variety of dissimilar diseases collected, I cannot say arranged, under the same general title. The error has has descended to us, and even in Dr. Cullen's Nosology we find diseases of arteries, veins, glands, tendons, joints, and bones, brought together under one order, and designated by the same name of *tumours.* Some of these also are merely enlargements of natural parts; whilst others are entirely new productions, having no existence in the original composition of the body. We have, I believe, sufficient knowledge of the nature of these diseases to class them more scientifically; and as this has not yet, as far as I know, been done, I shall endeavour to supply the deficiency,

"In the definition which I mean to give of tumours, I shall trespass as much against the usual import of the word, as nosologists have hitherto done in their classifications against the nature of the disease. For I shall restrict the surgical signification of the word tumour to such swellings as arise from some new production, which made no part of the original composition of the body; and by this means I shall exclude all simple enlargements of bones, joints, glands, &c. Many enlargements of glands are however included in the definition, as they are found to be owing to a tumour growing in them, and either condensing the natural structure, or causing the absorption of the original gland. Sometimes also the disease of the gland seems to produce an entire alteration of structure in the part; the natural organization being removed, and a new-formed diseased structure substituted in its stead. In either of these cases the disease of the gland is designed to be included in the definition; and the practical remarks which follow will equally apply to the same kind of diseased structure, whether it exists separately by itself, or occupies the situation of an original gland. The structure of tumours is also a part of morbid anatomy which deserves to be examined; since (as it did not come within the scope of the undertaking) it has not been fully discussed by Dr. Baillie in his very valuable treatise on that subject. Yet as he has given representations of glandular parts enlarged by a diseased structure of an entirely new formation; so I shall have the advantage of referring the reader to his accurate and expressive representations of some of those appearances which it is my purpose to describe. There is an observation of this judicious and accurate writer which I shall take the liberty of inserting, since it justly appreciates the degree of utility of investigations like the present: he observes, 'That the knowledge of morbid structure does not lead with certainty to the knowledge of morbid actions, although the one is the effect of the other; yet surely it lays the most solid foundation for prosecuting such enquiries with success. In proportion, therefore, as we shall become acquainted with the changes produced in the structure of parts from diseased actions, we shall be more likely to make some progress towards a knowledge of the actions themselves, although it must be very slowly."

Nothing can be more just than these remarks, but we cannot help wishing the definition had been more pointed. If these tumours, which are found to grow in glands, are new formed parts, there could be no occasion for the exception. If a new formed diseased structure is substituted instead of the original organization of the gland, the same objection seems to occur. However in this respect, Mr. Abernethy is more culpable in his arrangement than in his definition. By subsequent passages we find, that in all these cases of tumours he conceives an economy is established for the support of themselves different from, if not independent of, original formed parts; at least such seems the case by the following passage. "Thus an organized concrete (coagulated blood) becomes a living tumour, which has at first no perceptible peculiarity as to its nature, though it derives its supply of nourishment from the surrounding parts; it seems to live and grow by its own independent powers; and the future structure which it may acquire seems to depend on the operation of its own vessels. When the organization of the gland seems changed into that unnatural structure, which is observable in tumours, it may be thought in some measure to contradict those observations, but in this case the substance of the gland is the matrix in which the tumour is formed."

"The structure of a tumour," continues our author, "is sometimes like that of the parts near which it grows. Those which are pendulous into joints, are of a cartilaginous or osseous fabric; fatty tumours frequently form in the midst of adipose substance, and I have seen some tumours growing from the palate, and having a slender attachment, which in structure resembled the palate. Sometimes, however, they do not resemble in structure the parts from which they grow. The instance just mentioned, of the pendulous portion of fat growing from the peritoneum, will serve as an instance: the vessels which had shot into it, made the tumour into fat, whilst the neck was of a fibrous and vascular structure. I have seen osseous tumours unconnected with bone or periosteum; and indeed, in general, the structure of a tumour is unlike that of the part in which it is produced. Therefore we seem warranted in concluding, that in many cases the nature of the tumour depends on its own actions and organization; and that, like the embryon, it merely receives nourishment from the surrounding parts.

"If, then, the coagulable part of the blood be from any cause effused, if the adjacent absorbents do not remove it, and the surrounding vessels grow into it, the origin of a tumour may be thus formed. It may be right to reflect a little on the causes which may occasion a deposition and consequent organization of the coagulable part of the blood; as such reflections throw light on the nature and growth of tumours, and lead to the establishment of principles, which are applicable to tumours in general. The deposition of the coagulable part of the blood may be the effect of accident, or of a common inflammatory process, or it may be the consequence of some diseased action of the surrounding vessels which may influence the organization and growth of the tumour.

"In the former cases, the parts surrounding the tumour may be considered simply as the sources from which it derives its nutriment, ment, whilst it grows apparently by its own inherent powers, and its organization depends upon actions begun and existing in itself. If such a tumour be removed, the surrounding parts, being sound, soon heal, and a complete cure ensues. But if a tumour be removed, whose existence depended on the disease of the surrounding parts which are still left, and this disease be not altered by the stimulus of the operation, no benefit is obtained: these parts again produce a diseased substance, which has generally the appearance of fungus, and, in consequence of being irritated by the injury of the operation, the disease is in general increased by the means which were designed for its cure. It appears therefore that in some cases of tumours, the newly formed part alone requires removal, whilst in others the surrounding substance must be taken away, or a radical cure cannot be effected.

"There is yet another circumstance deserving attention, before I proceed to the particular consideration of the subject; which is, that a tumour once formed, seems to be a sufficient cause of its own continuance and increase. The irritation which it causes in the contiguous parts, is likely to keep up that increased action of vessels which is necessary to its supply; and the larger it becomes, the more does it stimulate, and of course contribute to its own increase."

These remarks lead our author to some reflections on the mode of cure in the early stage of tumour. These are by topical bleeding and cold applications. After the increased action is thus subdued, he proposes stimulant remedies to promote absorption. But if these tumours have really an economy of their own, we cannot easily conceive how application applied to the neighbouring parts can produce any lasting effect.

After these remarks, Mr. Abernethy enters on his division, and still confining the word to substances which made no part of the original structure of the body, he denominates the first genus from its firm and fleshy feel, *sarcoma* or *sarcomatous tumour*. This genus, he observes, contains many species. Those enumerated by our author are, *Common vascular or organized sarcoma*, *adipose sarcoma*, *pancreatic sarcoma*, *mastoid or mammary sarcoma*, *tuberculated sarcoma*, *medullary sarcoma* (sometimes called soft cancer), *carcinomatous sarcoma*. For the description of these we must refer our reader to the work, where he will find them illustrated with cases.

The next *genus* of tumours under the *order* of local diseases is *encysted tumours*. Though only one species is mentioned, yet it seems to comprehend so many varieties as might, we think, have authorised some divisions, if not into species, at least into varieties. Under the cases by which this genus is illustrated, we have the word wen more than once. We sincerely wish a precise meaning were given to the word, or that we were informed, that by the term is meant a tumour of any of the above description.

( To be continued. )

*An Account of the Discovery and Operation of a new Medicine for Gout, Second Edition, with many Additions, and Testimonies of Persons of the first Respectablity.* By A. Welles. 8vo. pp. 212. London, 1804.

It has been observed and acknowledged for two thousand years, that persons who have suffered repeated paroxysms of gout, *very rarely* became free from it during the remainder of their lives. This constitutes, in medical language, an *incurable* disease. There is no doubt that many persons, who have been attacked in the prime of life by this *sæva regina dolorum*, have, by means of regimen, remained free from any repetition of their sufferings for several years; and probably a large majority of such subjects might enjoy a similar exemption, if they possessed sufficient resolution to adhere strictly to the plan prescribed. The gout, however, notwithstanding the most rigid observance of regimen and temperance, will return at some time or other. These facts have been proved by sufficient time, observation, and experience. Another opinion, highly injurious to the advancement of medical science, has unfortunately sprung out of the former. It is, that the gout is not only incurable, but that every attempt to mitigate the sufferings of the patient is attended with great danger. This opinion has paralysed the faculty of medicine, and opprobriated the profession for two hundred years at least. But the liberal, enlightened, and correct Heberden has given a testimony, which justifies his juniors in exerting themselves to seek after more successful means of mitigating at least the miserable torments of arithritic sufferers. The valuable communication from Dr. Adams, contained in this Number, p. 141, on the efficacy of bark in relieving gout; the extract of aconite and cicuta, the vapour bath, the antiphlogistic plan of applying leeches and cold to the parts affected, all which have appeared in previous Numbers of our Journal, prove that the possibility of safely mitigating the tortures of gout is established beyond controversy. The author of the pamphlet before us has introduced a *new* article into the *materia medica*, which, we believe, will be a valuable addition to it.

The exhibition and effects of this vegetable production have been witnessed by a considerable number of respectable medical practitioners, some of whom have used it in their own cases; and the testimonies of the noblemen and gentlemen who have received unequivocal relief from the use of it, can leave no doubt in our minds that the proposed remedy merits particular attention, both from the profession and the public at large But here a question obtrudes itself, how far any respectable practitioner is justified in using or recommending a medicine, with the preparation and composition of which he is not familiar? All chemists know, that *vegetable* preparations cannot admit of any *analysis* which may lead to the slightest knowledge of their medicinal properties; and a chemical analysis of *mineral* preparations can do no more than discover discover the ingredients; and we infer the virtues from the known analogy of similar preparations, which must be loose and unsatisfactory. The only rational means then by which the virtues of any proposed remedy can be investigated, are repeated and well observed trials of it in the human subject. Mr. W. has pursued this plan; and as far as three or four years experience can establish a medical fact, he has proved his medicine to be safe and efficacious.

---

*An Answer to Mr. Goldson; proving that Vaccination is a permanent Security against the Small-pox. By John Ring, Member of the Royal College of Surgeons in London.* 8vo. pp. 43. London, 1804.

It might be expected that Mr. Ring's vigilant zeal in the cause of vaccination would be roused by Mr. Goldson's pointed attack on the utility of the Jennerean discovery, of which we gave an ample detail in our last number. The subject is so extensively important, and the attention excited by Mr. Goldson's publication has been so considerable, that we think it necessary, without delay, to state the leading particulars of Mr. Ring's answer, and the objections which he urges against the validity of Mr. Goldson's statements. In this, as in the former case, we shall chiefly confine ourselves to a fair representation of the writer's arguments, and a few observations on their consistency, leaving our readers to make their own inferences from what is advanced. We may premise that Mr. Ring's defence of vaccination against Mr. Goldson's statement, turns upon two points; either that the vaccination was insufficient, owing to faulty matter, and irregularity in the progress of the vesicle; or that the subsequent symptoms produced by small-pox infection, were only such as the introduction of this poison might at any time produce on certain constitutions, however indisputably they might previously have gone through the disorder.

Mr. R. first attempts entirely to vitiate the source of vaccine matter at Portsmouth. This, as we mentioned in our last Number, was sent by the Sick and Hurt Board to Mr. Rickman at Portsea. Of this Mr. R. observes,

"Mr. Goldson thinks it a sufficient proof of the Portsmouth matter having been good, that it was sent thither by a public board: but he has not proved that it was not procured from some place, where the golden rule of Dr. Jenner for taking matter was disregarded; and where matter was frequently taken at so late a period, as to produce spurious pustules, and bring disgrace on the practice.

"As Mr. Goldson has adduced no evidence that the matter was originally good, so he has offered none to prove that it did not remain on the lancets long enough to suffer injury before it was sent to Portsmouth. This was the more likely to happen, when it was taken in the worst mode possible, that is, on lancets; and had two offices to go through; at either of which these lancets might have remained long enough to become rusty. Mr. Goldson himself justly observes, that the success of vaccination is easily defeated, either from the matter having been originally inefficacious, or from its being deteriorated, and suffering a decomposition by a variety of means.

"With the matter received from London, Mr. Rickman inoculated five marines; and, with matter taken from the arm of one of them, he inoculated Clarke, whose case was communicated to the Committee of the House of Commons. This man, it was said, afterwards had the small-pox; and it is therefore an object of the first importance to ascertain, as far as possible, whether he ever had the cow-pock.

"In order to form a proper judgment in this instance, it ought to be recollected, that the matter issused from a doubtful source; that it was not taken till the 11th day, by which time it has often lost much of its virtue, and is apt to produce a spurious pustule; and that the only witnesses of its effect were persons, who had not the least pretensions to any knowledge or experience in the practice. It is, therefore no wonder the House of Commons considered this case as of no weight, when placed in opposition to the strong evidence brought forward by Dr. Jenner. And again,

"Mr. Goldson tells us, that in the course of his experiments, Mr. Rickman *soon found the matter run rapidly into a purulent state after the eighth day.* No stronger proof can be given that it was not good. This was the source of the matter first used by Mr. Goldson, and other gentlemen in the neighbourhood of Portsmouth."

In the usual progress of the vaccine vesicle, when no local injury happens to the arm, the contained fluid is at no time *purulent*, but, on the contrary, quite limpid to the last day that it can be obtained. This circumstance affords some ground of suspicion as to the efficacy of the matter originally employed; at the same time it is proper to state, that if Mr. Goldson's account be correct, vaccine inoculation from the same source, has produced, in numerous instances, the perfect disease, and has proved a complete prophylactic against repeated exposure to small-pox. Allowing the facts, the contradiction can only be explained by admitting the possibility of both perfect and imperfect vaccination from a vitiated source, under similar circumstances, and in each case with an entirely regular progress of the inoculated vesicle.

The case of Mr. Grant's child comes next under consideration. It was inoculated not with *Portsmouth matter*, but by Mr. Paytherus in London. No doubt is thrown on the genuineness of the matter here employed, and the perfect vaccination of the child. The eruption, which took place on the night between the sixth and seventh day, Mr. Ring attributes entirely to the cuticular inflammation produced by the variolous poison, and which, as Dr. Jenner has acutely remarked, is excited much more speedily in constitutions previously variolated or vaccinated, than where the poison poison produces the true small pox; and hence the rapidity of this inflammation is a pretty sure criterion of the constitution being secure from genuine variola. Mr. R. also points out, in the relation of this case, a small difference between Mr. Goldson's narration and that of Mr. Grant, the father of the child, in the time of the accession of the first symptoms of fever. On the evening of the seventh day the child was attacked with rigor and fever, and on the following morning a few eruptions appeared. On this Mr. R. remarks,

"As to the rigor, it is a common effect of suppuration; and the small pimples which appeared the next day were, in all probability, nothing but a miliary eruption. This eruption, it is well known, is the natural consequence of a hot regimen; and, in the present instance, there was a hot regimen with a vengeance.

"First, the child was rubbed before a good fire; then recourse was had to flannel and warm Madeira; and, lest any one stimulus should be wanting, an anodyne, as it is called, which commonly contains that powerful stimulus, opium, was administered by Mr. Goldson. With such an accumulation of heat, it is no wonder there were a few eruptions; it is rather a wonder the child was not covered with eruptions from head to foot.

"The small pimples which appeared, and caused such a terrible alarm, did not suppurate; but, in three days time, were covered with a warty scurf, which was rubbed off the following evening. If this is the small-pox, it is a sort of small-pox never heard of till now."

Mr. Ring proceeds to shew, by very satisfactory evidence, that the production of a local variolous pustule by inoculation after small-pox, occasionally of fever, and of a slight crop of pustules, is by no means an uncommon circumstance, and that the reason why we have had no more instances of such occurrences after variolous inoculation is, that the confidence in the efficacy of small-pox inoculation has been so complete, that few persons would give themselves the trouble of re-inoculation.

Having thus impeached the character of *Portsmouth matter* on the one hand, and shewn the possibility of mere cuticular inflammation by small-pox matter producing constitutional affection on the other, Mr. R. passes over the rest of Mr. Goldson's cases (which are all those inoculated by Mr. G. himself, and by Mr. Weymouth of Portsea) with very slight notice: either the vaccination was imperfect, or the subsequent disease was not genuine constitutional small-pox. One observation, however, requires some comment: in Mr. G's third case, the child, after supposed satisfactory vaccination, caught the small-pox, not by inoculation, but by casual contagion. Of course, cuticular inflammation is here out of the question, and Mr. R. therefore denies the genuineness of the vaccine inoculation. To prove it satisfactory, Mr. Goldson states, that two years afterwards the child slept with another child in small-pox for several days, wore the same night-

 cap, cap, and, in short, was as much exposed to contagion as possible.' On this Mr. Ring makes the following remark.

"It is too ridiculous to conclude, that, because a child did not catch the small-pox when she wore an infected night-cap, she could never catch it while she lived; and, unlesss gentlemen can bring better proofs than these of a temporary security arising from vaccination, they had better put on their own night-caps, and go to bed.

"The truth is, that it is no uncommon circumstance for a person to catch the small-pox who has resisted it before; and even resisted it for a long time, in every form, and every degree of exposure. Many a parent, after attending several children successively in the small-pox, and arriving at an advanced period of life, has at length fallen a victim to that disease."

There is nothing very *ridiculous* in the supposition, that a child who has once resisted such a degree of small-pox contagion should be ever after secure from the disease: no practitioner after such a test would hesitate in asserting the *high probability* of future security throughout life; and we appeal to Mr. Ring's candour, whether he would not have used this circumstance as a most powerful argument for the permanent efficacy of vaccination to any of his own patients, or to this very case, had not the event turned out so contrary to expectation. That we are not mistaken in this assertion, let us judge Mr. Ring from his own words. In his letter to Mr. Grant, here inserted, Mr. R. observes, "I have long since discontinued the practice," (of re-inoculating vaccinated patients with small-pox matter) "satisfied with exposing my patients, after vaccination, to the natural infection of the small-pox. This, which, on my assurance of safety, is submitted to with the utmost confidence, has removed all remaining doubt from the parents of the children vaccinated by me, who now amount to about two thousand five hundred. Many of these have been put into bed with persons labouring under the confluent small-pox, or wrapped up in sheets just taken off from the beds of variolous patients, with impunity." Will Mr. Ring, therefore, point out how many times of safe exposure to small-pox contagion is necessary to render the idea of permanent security probable, or where it ceases to be ridiculous? Nevertheless, we are far from supposing that this single case of Mr. Goldson's is to outweigh the strong evidence of the permanent efficacy of *perfect* vaccination; and we think the following testimonial one of the most valuable parts of Mr. Ring's pamphlet, because it is simple indisputable evidence:

"Three children of Mr. Henry Jenner, inoculated five years ago, have since been repeatedly inoculated with variolous matter, and exposed to the infection of the natural small-pox in its worst form, every year up to the present time, without catching the disease.

"Pead,

"Pead, vaccinated by Dr. Jenner more than six years, and Phipps, his first patient, vaccinated by him more than eight years ago, have been frequently put to the same tests with impunity. In the spring of the present year, they were inoculated for the small-pox with matter in the most active state; but they resisted infection.

"These patients were all vaccinated with matter from the human subject. Time, therefore, *has decided the question*, whether cow-pock matter degenerates in the human subject, and decided it against Mr. Goldson.

"Instances out of number might be adduced, if necessary, in support of this position. The cow-pock is transfered by the milkers, not only from one cow to another, but also from one farm to another; which could not be the case, if the matter lost its virtue after the first remove from the cow. One instance lately occurred, which furnishes an incontrovertible proof, that vaccine matter, whether generated in the cow or in the human subject, is the same. A woman lately applied to Dr. Jenner, who had the cow-pock when a child. She caught the disease by handling the rags which came off from her sister's fingers. Dr. Jenner inoculated her for the small-pox; but she resisted the infection.

"I shall here insert two other cases; with which, as well as almost every thing else relative to the subject of vaccination, I have great reason to believe, both from the tenor of his pamphlet, and from intelligence I have received, Mr. Goldson and his friends are totally unacquainted. The first case, which was published by Dr. Barry, is as follows: A gardener, who lives with a gentleman of Dr. Barry's acquaintance, infected himself with the cow-pock, by rubbing himself against another person who had received the infection from the cow, from a conviction that it would preserve him against the small-pox. This happened several years ago. Since that time he has often voluntarily exposed himself to the infection of the small pox, and even lain in the same bed with his children, when they were covered with it, but never caught the disease.

"The other case was published by Mr. Creaser. It was communicated to him by Mr. White, of Landsdown Place, Bath. About twenty-three years ago, John Bright, a labouring man, whom Mr. White sometimes employs, lived at a farm. His fellow-servant, who had the cow-pock, communicated the distemper to him in a frolic, by means of a scratch on the hand. He has since been repeatedly inoculated for the small-pox. He has also had the disease in his family, and been exposed to it under its most malignant form, but still escaped infection."

A very pointed evidence from the Vaccine Institution of Edinburgh is also given in favour of the undiminishing power of vaccination through three, four, and even five years.

What then remains to be done by the advocates of vaccination? Mr. Ring, it will be seen by the following passage, is for standing aloof aloof from all attempts by experiments at doing away the impression made on the public mind by Mr. Goldson's statement.

"Mr. Goldson solicits the Vaccine Institution to make fresh experiments, in order to decide a question which is long since decided. What institution he means may, like a considerable part of his observations, admit of a doubt. Whether there be any vaccine institution that will so far disgrace the cause, as to repeat such experiments at Mr. Goldson's request, and whether there be any vaccine institution that would not disgrace itself by such an act, I shall not presume to determine; but the Royal Jennerian Society have passed a resolution, that Mr. Goldson's pamphlet does not, at present, require any notice from them. I trust they will still adhere to that resolution:

"Nec deus intersit, nisi dignus vindice nodus.

"It does not require a hundred able heads to plan, nor a hundred able hands to execute, the simple task of putting vaccine patients to the test. It is what any head, however weak, can plan, and any hand, however unskilful, can execute.

"But when we consider what a vast number of persons have been vaccinated in this metropolis, and are daily exposed to the danger of catching the small-pox in the natural way, we cannot but deem it a work of supererogation to try such experiments again if they are innocent, and a crime to try them again if they are attended with danger."

Probably the impression made by Mr. Goldson's cases will subside, and the appearance of contravening evidence will sooner or later be entirely lost in the daily accumulating mass of testimony in favour of vaccine inoculation pouring in from every quarter of the globe; but why should any of the friends of this practice hesitate immediately to institute those experiments which may speedily put an end to the controversy? Why should they think it a disgrace in such a cause, and with such reliance on success, to convince the doubtful, silence the malicious, give confidence to the anxious, and hasten the great object of all their endeavours, the annihilation of the small-pox from the face of the globe? Some consistency too should be preserved with regard to the alleged security of the proof-trial of subsequent variolous inoculation. It has been hitherto an argument of no small weight in the hands of the friends of vaccine inoculation, "it can at least do no harm; first give your child the benefit of the cow-pox and you may then safely try him with the small-pox inoculation, when you will be convinced by the result how perfect is the security which vaccination produces." But if, whilst a considerable part of the public is still averse, or but coldly disposed towards vaccination, the only palpable proof of its efficacy is wantonly held out as full of danger, and not lightly to be resorted to, how much will not such ill-judged zeal injure the cause it strives to serve! What is this mighty danger of variolous inoculation? Mr. Ring himself acknowledges to have performed it upon eleven hundred persons after vaccination without without any untoward accident, and many more hundreds, or thousands of instances, equally innoxious, may be added to the list. Even the cuticular inflammation and consequent symptoms, though as severe as in Mr. Goldson's cases, produce no more than a day or two of indisposition, and an eruption of a few pimples. Mr. Ring will surely not magnify *these* into a disease of very great moment; and the only cases of danger which he brings are two from Buchan, which, however authentic, are little adverted to, on account of the extreme rarity of the occurrence.

The present pamphlet contains several other desultory remarks, for which we shall refer our readers to the original; and here we should close our account, if it were not incumbent upon us to repel an accusation which the Author has thought proper to bring against the manner in which we noticed Mr. Goldson's pamphlet in our last number. For this we must beg our reader's indulgence a short time longer.

We are first accused of inconsistency in exculpating the medical men of Portsmouth from the charge of indifference to professional improvement, at the same time that we urge the local advantages for scientific information of a situation so near to the metropolis, and so constantly communicating with it. The truth seems to be, that if Mr. G's dates are accurate, the profession at Portsmouth can neither claim the merit of early exertions in vaccination, nor yet are they liable to the reproach of culpable neglect. The accusation was trifling, and Mr. G's defence weak.

We are next charged with mistaking the object of Mr. Goldson's dedication. As Mr. G. himself has committed the mistake, (an unaccountable one we acknowledge) we cannot pretend to reconcile his contradictions; but though sent to Salisbury Square, we must still suppose that the author meant to dedicate his book to the Vaccine Pock Institution, for this obvious reason, that Mr. G. addressed his Remarks to some Institution expressly for Vaccination, which had existed long enough to be able to undertake those experiments of reinoculation that he recommends. Accordingly, this respectable Society has taken Mr. G's dedication to itself, and has actually pursued the subject by the sure path of experiment.

As we have no desire to spare ourselves any of Mr. Ring's abuse, we shall insert the following.

"The article in question is one of the many in modern times, which makes us regret that the review of books is a t ade; that it is too often delegated to persons incompetent to the task, too often prostituted to the purposes of party, and too often made subservient to sordid gain. Such a practice cannot be reprobated too severely. On so important an occasion as this, no understrapper should be employed; no partial statement should be admitted; no puff, no misrepresentation, no compliment at the expence of truth, should be suffered to pass without animadversion."

It is our chief object in our monthly notice of new publications, to confine ourselves strictly to critical analysis, or rather, to state more more or less succinctly the leading contents, with or without occasional remarks on the propriety, originality, consistency, and candour of what is advanced. We have no apprehension of being accused of wilful misrepresentation of matter of fact; and as we make a point of giving the several author's own words, more or less condensed, in the most important statements, there is little danger of much accidental error. Mr. Ring accuses us of admitting Mr. Goldson's statements rather too readily. Now, as all that we have inserted of Mr. G's facts is almost in his own words, and given simply as his own cases, does Mr. Ring mean to charge us with having done *too much justice* to Mr. G.? or, would this enemy of partial statements, have had us suppress a part, and falsify the remainder?

No small portion of the offence we have given to Mr. Ring (we believe) is included in the following accusation.

"But it is puerile to pretend, that a medical man is the more competent to put his cow-pock patients to the test, in consequence of his belonging to a Society. Any individual is qualified for that undertaking; and it is easy to collect witnesses of his proceedings in any town or village in the kingdom. There are several *individuals*, members of the Royal Jennerian Society, besides Dr. Jenner himself, whose practice has been far more considerable than that of the Vaccine Pock Institution, both in extent and duration. It is therefore an insult to deny, that they have it as much in their power to institute further experiments on the subject; as it is a mockery to affirm, that no party is so fit to decide the point, as that which furnished the matter, and of course is interested in the question."

Mr. Ring is not in all cases an enemy to misrepresentation, or he would not have made us express so very absurd an opinion, that a medical man is more competent to reinoculate his patients when belonging to a society than when standing singly on his own experience; or that any "individual member of the Royal Jennerian Society, whose practice has been far more considerable than that of the Vaccine Pock Institution," would not perform a most acceptable service to vaccination, by instituting farther experiments on his own patients; but we do maintain, that the Vaccine Pock Institution *is* particularly called upon in this case, if it furnished the *Portsmouth matter*, and if, from the duration of the Society, it is able to meet the call in a satisfactory manner. Had a similar imputation been thrown on Mr. Ring's matter, would he not have thought himself peculiarly interested in vindicating its genuineness? and though a professed enemy to puffing, would he not, at least on such an occasion, allow himself to state the extent and duration of his own practice, as additional motives for his exertions? Lastly, let Mr. Ring remember, that the pen which is truly *prostituted for the purposes of party*, is that which, without bringing forward the least shadow of evidence, stoops to personal defamation of his opponents, and throws out unequivocal insinuations of sordid and base base motives of private gain acting in direct hostility to the efforts of philanthropy and public spirit. The accusation of sinister motives is easily made, and as easily retorted ; but the best friends of the Jennerean discovery will the most lament that it is defended by such weapons.

